# Women Who Are Not Trained Nurses

**Published:** 1920-02-14

**Authors:** 


### THE VOLUNTARY HOSPITAL AND THE NURSE.

### Women who are not Trained Nurses.

I WAS greatly struck by a reply to a correspondent's inquiry that appeared in a recent issue of THE HOSPITAL. There was nothing contained in the answer but that which is true, and of which I was already aware. Yet a plain, blunt statement of fact sometimes causes a shock even to those who know.

The question was simple—" How can I train as a health nurse? " And the reply was equally simple—" Apply for particulars to the Battersea Polytechnic, the National Health Society, and the Royal Sanitary Institute." In these few words, so seemingly innocent and natural, is the announcement of a public scandal, and if the people who raised such a din about State registration in the past had the nurse's economic welfare at heart the public would not have to be referred to the institutions named for the special training necessary for a health visitor. In previous articles in the journal I pointed out that according to the Minister of Health, and the literature which issues from his Department, the ideal health visitor is a trained nurse, or a certified midwife, who has undergone a supplementary course of training and instruction. There are a hundred reasons why the voluntary hospitals, which owe their very existence to the nurse, should provide such training. Not the least obvious of these are the hospital interests. It is good for a hospital to have a constant flow of new blood, which would be supplied by its trained nurses finding a wider field of employment. It is also good for a hospital to be able to avail of the highest standard of probationers anxious to enter for training. These considerations alone, one would think, ought to be sufficiently strong to convince hospital committees of the necessity of arriving at a working agreement with the Ministry of Health. I am, of course, aware that the voluntary hospital is fighting for its life, and that the financial problem is paramount. There can be little doubt, however, that the welfare of the nurse is intimately associated with the welfare of the hospital. Nurses as a body are potential to influence a great volume of public opinion, and they would do so, without being asked, if they knew and realised that they had something like a proprietary interest in their success and prosperity I regret to have to state that, after much careful consideration, very few, if any, of our hospitals have played the game so far as the nurse is concerned. They have exacted from her the maximum of work at a minimum of remuneration. We have it from the chairman of the London Hospital in the year of grace 1920 that a twelve-hour day is reasonable for a nurse, and not tiring.

Now, what is happening? Just this, that after bearing the burden and heat of this trying day year in and year out for a whole generation—after the stress and strain of war, which the trained nurse shared to the full, outside institutions are busy preparing women who are not trained nurses to filch from them what is their natural heritage. I do not think I am using any exaggerated language when I describe this as a public scandal. And I cannot saddle the blame on the Ministry of Health. I made a careful study of their announcements in this particular matter, which amounted in a few words to this, that the best candidates for employment are trained nurses, that they are extremely anxious to have them, and that their curriculum of special supplementary training was open to revision and compromise. So far as I am able to ascertain, the hospitals have offered no response, and so the Ministry is going out into the highways and byways to find the trained assistance that is necessary for the effective carrying out of the national health programme.

This attitude on the part of the hospitals is not alone shabby and ungrateful, but narrow and shortsighted. If the different Boards of Governors could see it, it is as much opposed to their own institutional interests as it is negligible of the nurse's. The sick-poor clamour at their gates, and the difficulty of finding ways and means is great. This may be so, and is. But the way of salvation does not lie in self-contemplation. Many a man in misfortune over which he had no control has helped to his own downfall by what the lawyers call contributory negligence. I entertain no shadow of doubt that the voluntary hospital could make no more effectual or louder call on the public for sympathy and support than through its nursing staff. Has this been done? I think the answer is to be found in Dr. Addison's "scullerymaid" comparison.

IERNE.

